# Single-cell RNA Sequencing Uncovered the Involvement of an Endothelial Subset in Neutrophil Recruitment in Chemically Induced Rat Pulmonary Inflammation

**DOI:** 10.7150/ijms.67806

**Published:** 2022-03-28

**Authors:** Hong Huang, Ying Yang, Tingting Song, Yongfeng Yang, Yihan Zhu, Zhiqiang Liu, Li Li, Xuedong Wang

**Affiliations:** 1 Institute of Clinical Pathology, Key Laboratory of Transplantation Engineering and Immunology, Ministry of Health, West China Hospital, Sichuan University, Chengdu, Sichuan, China.; 2 Institute of Respiratory Health, Frontiers Science Center for Disease-related Molecular Network, West China Hospital, Sichuan University, Chengdu, Sichuan, China.; 3 West China Second Hospital, Sichuan University, Chengdu, 610041 P. R. China & Key Laboratory of Birth Defects and Related Diseases of Women and Children (Sichuan University), Ministry of Education.

**Keywords:** pulmonary inflammation, single-cell RNA sequencing, endothelial cell, neutrophil recruitment

## Abstract

There is growing support for the notion that chronic inflammation contributes to lung tumorigenesis, but the molecular and cellular basis underlying the protumorigenic effects of inflammation remains to be explored. 3-Methylcholanthrene and diethylnitrosamine were intratracheally instilled into rats to induce multistep lung carcinogenesis, and the presence of pulmonary inflammation was observed in addition to precancerous lesions. By leveraging single-cell RNA sequencing, we sought to unravel the mechanism underlying the inflammatory process at a higher resolution. A total of 14 cell types were identified in chemically treated and control rats. Chemical intervention introduced heterogeneity in cell type composition and gene expression patterns. Nonimmune cells were found to be the most affected, and two subpopulations of endothelial cells with diverse roles were defined. Car4-high endothelial cells were mainly responsible for angiogenesis, whereas Car4-low endothelial cells were involved in neutrophil recruitment, and adhesion between Car4-low endothelial cells and neutrophils was verified in inflamed tissues. Our work unveiled the intricate process of pulmonary inflammation at the single-cell level and characterized a proinflammatory subpopulation of endothelial cells involved in neutrophil recruitment. The conditions provided by chronic inflammatory environment are prerequisites for neoplastic progression. Targeting the specific subsets or processes defined herein holds promise for the early prevention and therapeutic intervention of lung cancer through the manipulation of angiogenesis or the inflammatory response.

## Introduction

Inflammation is frequently described as a hallmark of malignancy 1, 2. In fact, persistent chronic inflammation may affect susceptibility to carcinogenesis through some known mechanisms 3. Initiation of the lung immunoinflammatory response, which can be induced by exposure to toxins, asbestos, silica particles, tobacco smoke, or other irritants, may lead to an increased risk of lung cancer 4. Inflammatory cells promote the premalignant transformation of bronchial epithelial cells and their gradual development into precancerous lesions 5; such inflammatory cells with considerable roles in lung carcinogenesis include innate immune cells (e.g., neutrophils, macrophages, monocytes, and dendritic cells), adaptive immune cells (e.g., T lymphocytes and B lymphocytes), and tissue cells (e.g., endothelial cells (ECs) and fibroblasts) 6. The leukocytes first recruited to sites of tissue injury or infection are innate immune cells, primarily neutrophils, whose recruitment is essential for the effective control of inflammation 7. The importance of this orchestrated process is exemplified in patients with leukocyte adhesion deficiency syndromes 8. The regulated recruitment of neutrophils begins with inflammatory stimulation, chemoattractant presentation and adhesion molecule expression, which evoke neutrophil signaling and arrest, followed by endothelial signaling, neutrophil migration, and eventually the recruitment of other types of leukocytes to the inflamed tissue 9. Under conditions of pulmonary inflammation, inflammatory cells regulate the microenvironment by releasing a variety of cytokines and chemokines 10, 11. Through the joint regulation of multiple adhesion molecules, an adhesion cascade is formed via a programmed mechanism. Therefore, exploring the functions and changes of inflammatory cells in the inflammatory microenvironment that may contribute to the occurrence of lung cancer is expected to provide instructive strategies for the early prevention and immunotherapy of lung cancer.

Intratracheal instillation of 3-methylcholanthrene (MCA) and diethylnitrosamine (DEN) has been demonstrated to induce various pathological phases of lung carcinogenesis in rats 12. In our study, pulmonary inflammation was detected in addition to precancerous microlesions during the establishment of the rat model. We are committed to exploring the mechanisms underlying cell-cell interactions and identifying transcriptional signatures in chemically induced lung inflammation by single-cell RNA sequencing (scRNA-seq). High-powered single-cell transcriptomics enables the investigation of cellular diversity and the illumination of cell interactions during disease development, providing insights into the molecular basis of inflammatory microenvironment 13. At single-cell resolution, we identified the Car4-low endothelial subset and elucidated the biological process that programs the specialization of this subset, thus providing evidence for its role in regulating neutrophil recruitment in pulmonary inflammation.

## Results

### Intratracheal instillation of MCA and DEN induced pulmonary inflammation in rats

**Figure 1:**
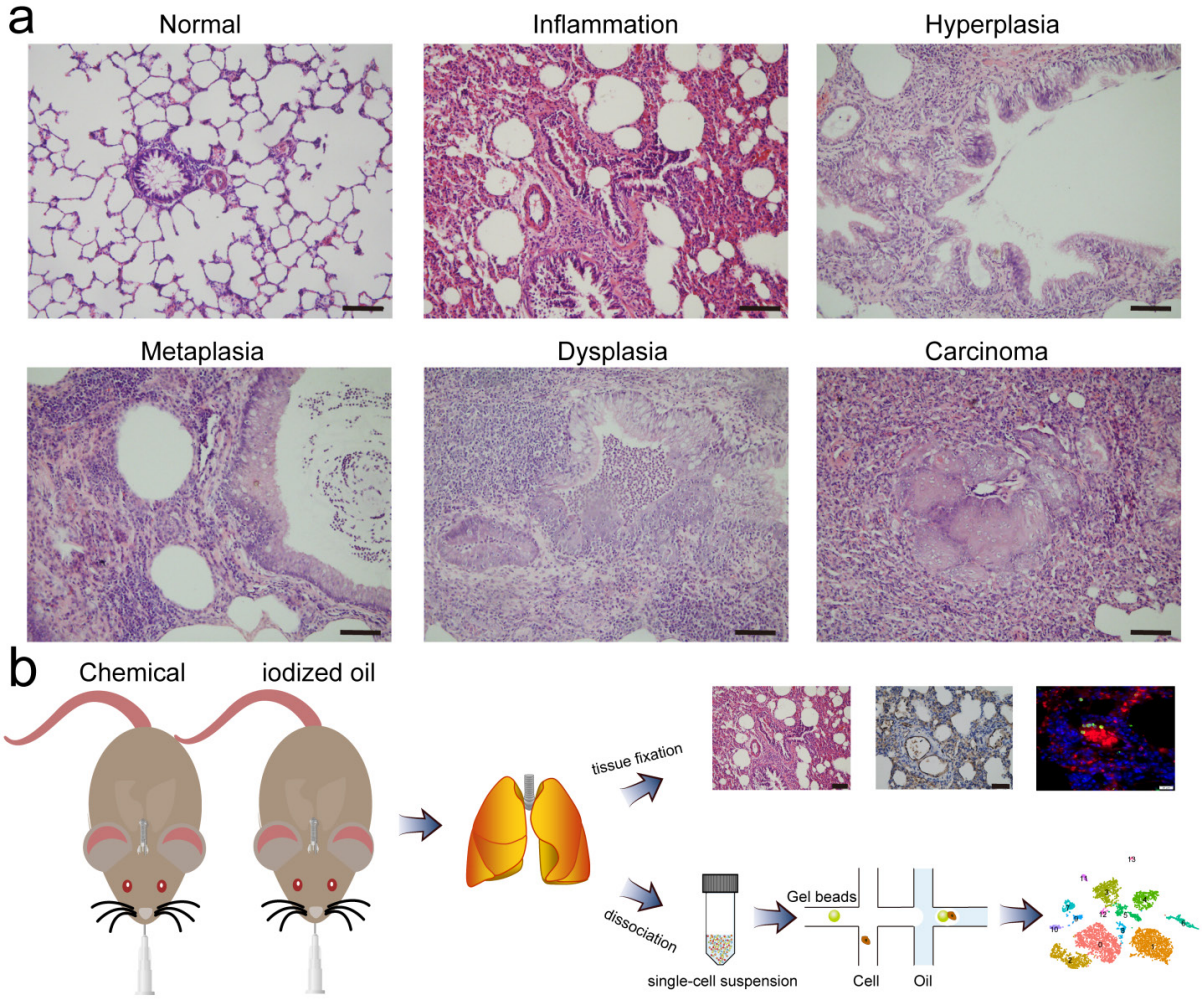
** Pathological classification and schematic diagram. a** Representative images of H&E staining of rat lungs treated with iodized oil (normal) or carcinogens (inflammation, hyperplasia, metaplasia, dysplasia, and carcinoma). Scale bar: 200 µm. **b** Flowchart overview of rat model establishment, single-cell RNA sequencing, and histological experiments.

During the exploration of multistep carcinogenesis in rat model, we found that infusion of carcinogens (MCA and DEN**)** into the trachea resulted in the formation of different pathological lesions, from hyperplasia, metaplasia, and dysplasia to lung carcinoma. In addition, the presence of pulmonary inflammation was observed. Typically, chemical intervention caused adhesion of the bronchial mucosal epithelium, and epithelial cells degenerated and became necrotic or even detached. The exudation and infiltration of blood cells and inflammatory cells were observed in the focal area of lung tissue. In the alveolar area, we found significant alveolar septal thickening and alveolar cavity shrinkage. Histological examination of the control group revealed no obvious pathological changes (**Fig. 1a**). Except for those used for histological classification, the remaining lung tissues were enzymatically dissociated and processed into single cell suspensions, which were then loaded into a droplet-forming microfluidic device for scRNA-seq. A schematic diagram of this study is shown in **Fig. 1b**.

### Clustering and cell type identification based on single-cell transcriptomes

**Figure 2:**
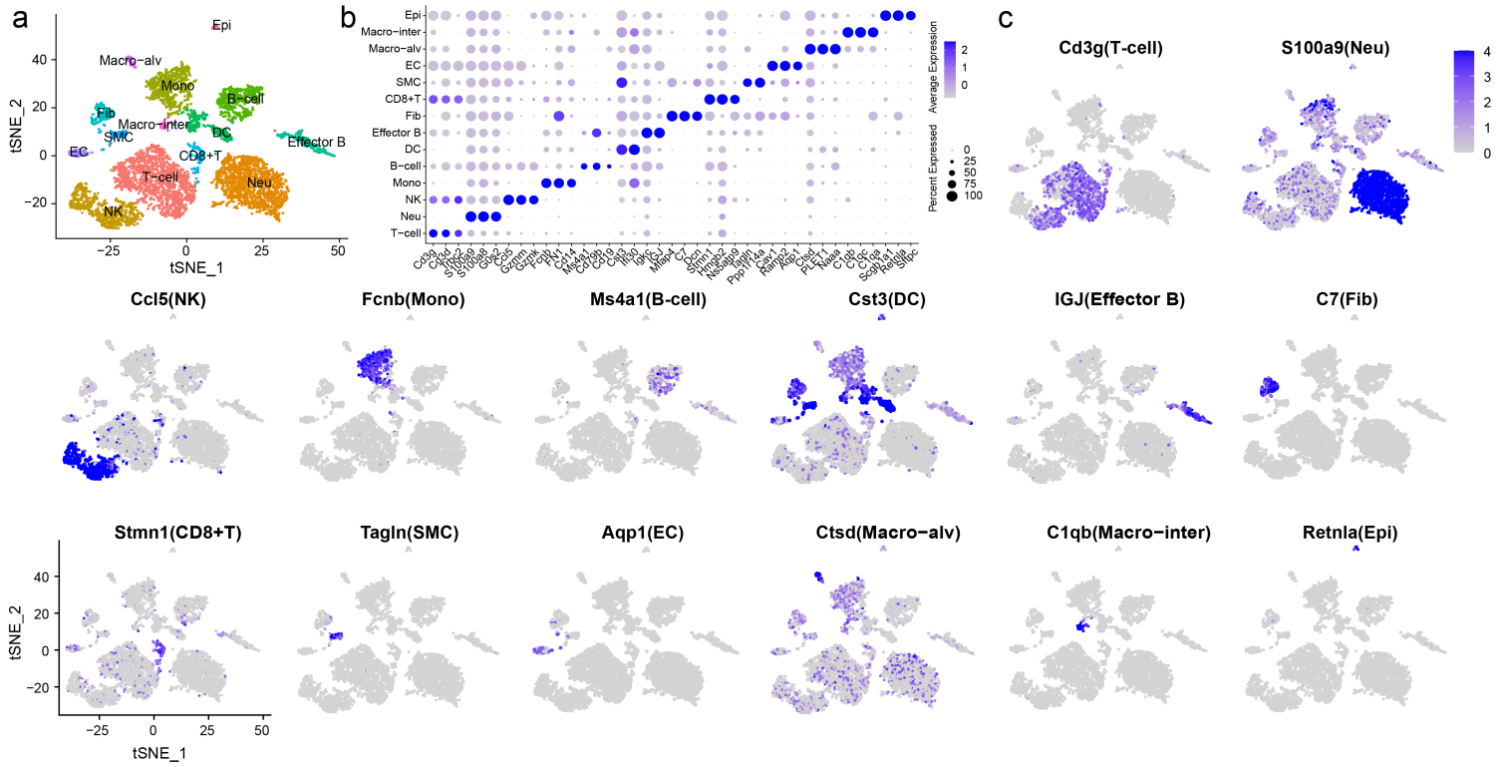
** Unbiased clustering resolved 14 cell types with distinct expression patterns. a** Unbiased clustering and cell type annotation visualized by t-distributed stochastic neighbor embedding (t-SNE) plot, with colors denoting cell types. **b** Dot plot of selected top marker genes for all cell types. **c** Feature plot of markers used to define each cell type. The color gradient represents the expression level. Epi: epithelial cell, Macro-alv: alveolar macrophage, Macro-inter: interstitial macrophage, EC: endothelial cell, SMC: smooth muscle cell, Fib: fibroblast, DC: dendritic cell, Mono: monocyte, NK: natural killer cell, Neu: neutrophil.

To elucidate cell type composition and transcriptional profile, we generated single-cell transcriptomes of samples from chemically treated and control rats. 2900 and 3530 cells, respectively, were obtained for subsequent analyses. The scRNA-seq data were first processed with Seurat R for quality control, normalization, batch effect correction, and clustering. By integrated analysis, clusters with fewer cells in one sample could be classified. Unbiased clustering was conducted using canonical correlation analysis (CCA) implemented in Seurat, which resolved 14 well-demarcated clusters visualized by a t-distributed stochastic neighbor embedding (t-SNE) plot (**Fig. 2a**). Then, the FindAllMarkers function was used to identify the feature genes of each cluster, and cell types were manually annotated according to the specific expression of canonical markers (**Fig. 2b, c**).

T cells featured high expression of Cd3g and Cd3d 14. Selected marker genes were also expressed in natural killer (NK) cells and CD8^+^ T cells. Multiple specific genes enabled the identification of neutrophils (Neu: S100a9, S100a8, and G0s2), NK cells (Ccl5, Gzmm, and Gzmk), and monocytes (Mono: Fcnb, FN1, and Cd14) 15-17. B cells showed high expression of Ms4a1, Cd79b and Cd19 18, 19. Cd79b was also expressed by effector B cells, which were characterized by the expression of IGJ and the proinflammatory factor Igkc. Dendritic cells (DCs) exhibited the expression of Ifi30 and Cst3 20. Other immune cells included CD8^+^ T cells, which expressed the marker genes Stmn1 and Ns5atp9, and notably, elevated expression of proliferation markers, including Pcna, Mki67, and Mcm2, was observed. Two distinct macrophage populations were identified, defined as alveolar macrophages (Macro-alv: Ctsd, PLET1, and Naaa) and interstitial macrophages (Macro-inter: C1qb, C1qc, and C1qa). Nonimmune cells displayed completely distinct gene expression patterns. Fibroblasts (Fib) expressed Mfap4, C7, and Dcn 21. Smooth muscle cells (SMCs) were identified by the marker genes Tagln and Ppp1r14a. ECs exhibited high expression of Cav1, Ramp2, and Aqp1 22, whereas epithelial cells (Epi) featured high expression of Scgb1a1, Retnla, and Sftpc. Scgb1a1 is a marker of Clara cells, and Retnla and Sftpc are markers of type II pneumocytes. Due to the limited number of captured cells, we failed to distinguish different subsets of epithelial cells. The gene expression patterns across clusters and the markers used for cluster identification are shown in **Fig. S1** and** S2**.

### Heterogeneities in cell type composition and gene expression were identified between groups

**Figure 3:**
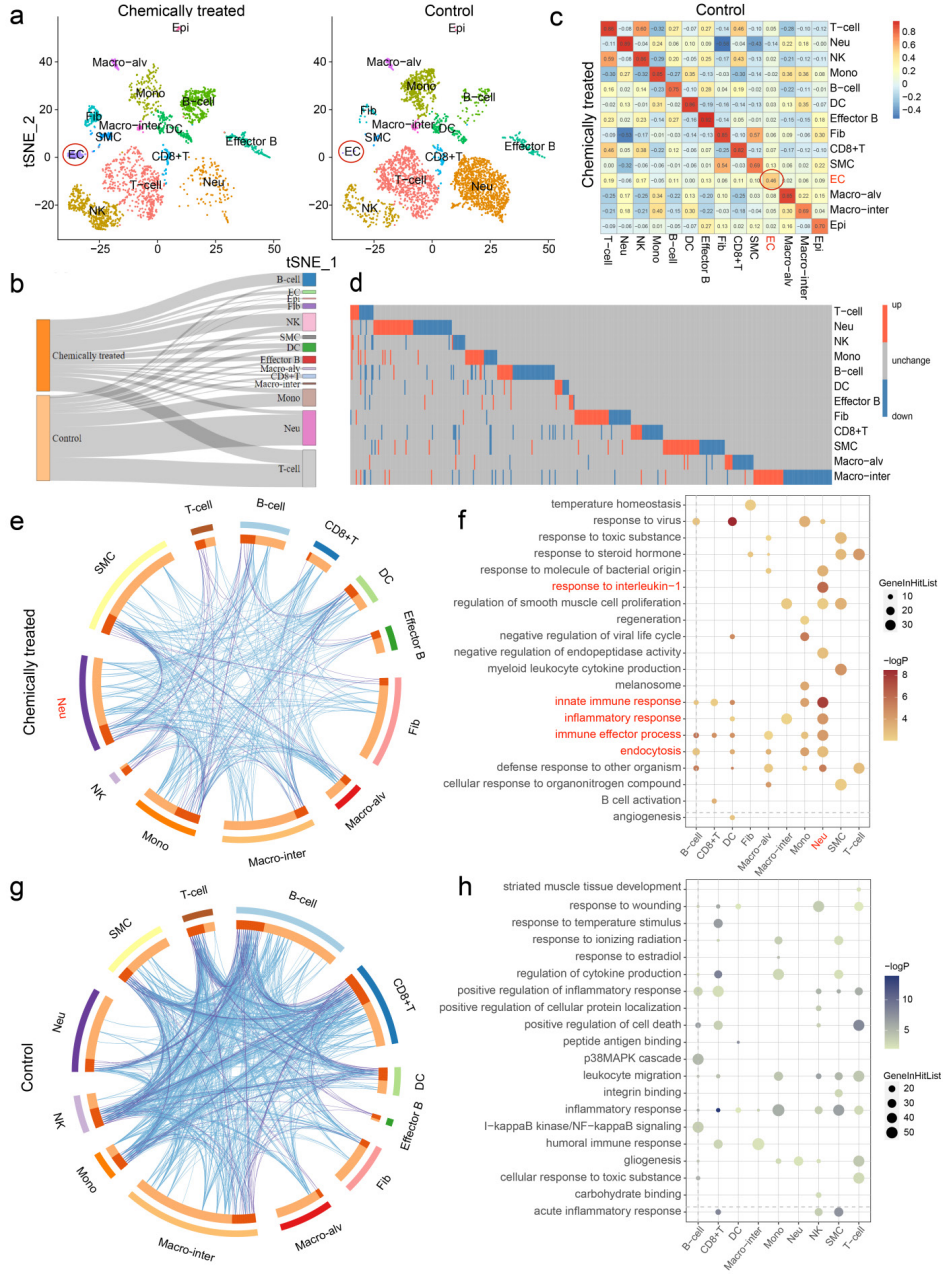
** Chemical intervention caused heterogeneity in cell type composition and gene expression. a** t-SNE plots of the separate clustering of chemically treated and control groups. **b** Changes in the relative proportions of each cell type are shown in the Sankey diagram. **c** Correlations of cell types between two groups. The color gradient represents the correlation coefficient. **d** Heatmap showing the distribution of differentially expressed genes (DEGs) across cell types (EC and Epi are not shown). Each row represents one cell type, and each column represents one gene. Red, upregulated; blue, downregulated; gray, unchanged. **e, g** Circos overlap by gene ontology (GO) terms using Metascape. **e,** Chemically treated; **g,** Control. Blue lines indicate functionally identical or related biological processes; purple lines indicate genetic overlap between samples. **f, h** Top 20 GO terms enriched by DEGs based on functional enrichment analysis. **f,** Upregulated; **h,** Downregulated. Epi: epithelial cell, Macro-alv: alveolar macrophage, Macro-inter: interstitial macrophage, EC: endothelial cell, SMC: smooth muscle cell, Fib: fibroblast, DC: dendritic cell, Mono: monocyte, NK: natural killer cell, Neu: neutrophil.

After determining the identity of each cluster, we next sought to investigate the cell populations that responded differentially in chemically treated and control rats. Indeed, clustering of the separate datasets yielded similar cell assignments to clustering of the integrated dataset, highlighting the robustness of the clustering results (**Fig. 3a**). Changes in the relative proportions of the cell types are comprehensively depicted in the Sankey diagram (**Fig. 3b**). The proportions of B cells and NK cells were elevated significantly in lung inflammation, indicating the presence of an immune response. Nonimmune cells were more affected by the intervention. ECs, Epi, Fib and SMCs were present in markedly higher proportions in the chemically treated group than in the control group. Moreover, the correlation coefficients of ECs, SMCs and Epi between the groups were relatively low, especially that of ECs (0.46) (**Fig. 3c**). The effect of carcinogens on gene expression was also evaluated. The differentially expressed genes (DEGs) revealed heterogeneity in gene expression between samples, and the DEG set for each cell type was considered unique (**Fig. 3d**). Despite changes in cell type proportions and correlations, a few DEGs were identified in ECs and Epi due to the limited amount of data (data not shown). To unveil the biological functions associated with the DEGs across cell types, Gene Ontology (GO) enrichment analysis was performed. Circos overlaps indicated functionally related biological processes, and heterogeneity in the genetic correlation among cell types was observed between samples (**Fig. 3e, g**). Differentially regulated GO terms for each cell type pertaining to the different treatments were identified, and the detailed results are shown in **Fig. 3f, h**. Typically, the upregulated DEGs for neutrophils were enriched in the innate immune response, response to interleukin-1, inflammatory response, immune effector process, and endocytosis, suggesting their crucial roles in rat pulmonary inflammation.

### Single-cell reclustering revealed two endothelial subpopulations with distinct roles

**Figure 4:**
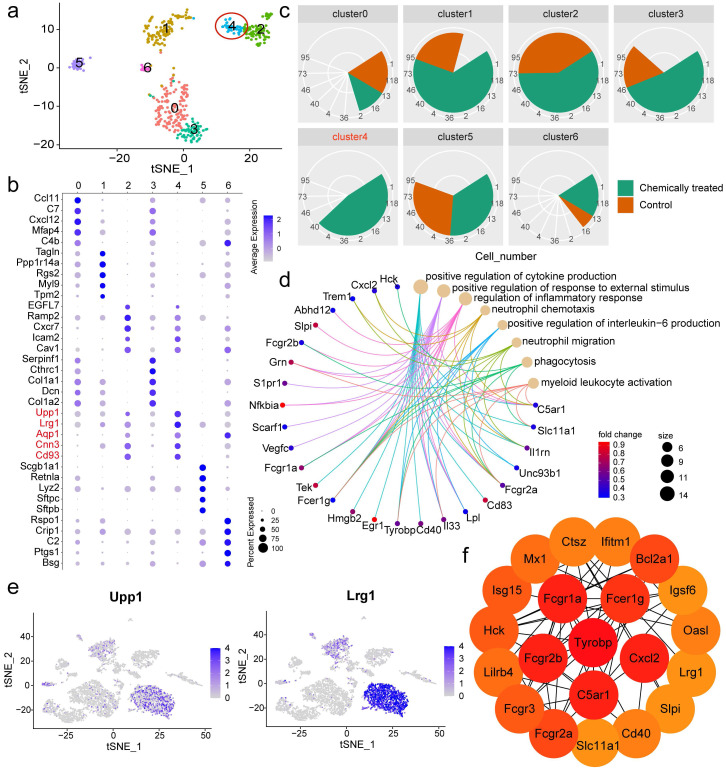
** Reclustering of nonimmune cells identified Car4-low ECs with specific functions. a** t-SNE plot of the reclustering of nonimmune cells. Colors denote different clusters. **b** Dot plot of the top 5 feature genes detected in each nonimmune cluster. **c** Comparison of the cell number corresponding to each nonimmune cluster between samples. **d** Representative biological processes enriched in Car4-low ECs, and related genes. Colors denote different GO terms. **e** Feature plots of Upp1 and Lrg1 across all cell types. **f** Hub genes identified in Car4-low ECs using Cytoscape software. The color gradient represents the importance of the genes in the cell subpopulation. EC: endothelial cell.

To further elucidate the heterogeneity within the nonimmune population, cluster analysis was performed again, and a closer inspection was conducted to identify marker genes in each subpopulation relative to the rest of the nonimmune population. A total of seven subclusters with diverse transcriptomic profiles were identified (**Fig. 4a**). The markers used to define each subcluster are displayed in a dot plot (**Fig. 4b**), and the feature genes of each subpopulation are listed in **Table S1**. Regarding the proportions of the subsets, cluster 4 was found to exist specifically in the chemically treated group (**Fig. 4c**). The aforementioned EC markers Aqp1 and Cnn3 allowed us to identify nonimmune cluster 4 as an EC subpopulation. To infer the potential functions of this cluster, functional enrichment analysis of its signature genes was performed. Representative terms were involved in positive regulation of cytokine production, positive regulation of response to external stimulus, regulation of inflammatory response, and neutrophil chemotaxis (**Fig. 4d**). The other endothelial subpopulation, cluster 2, played distinct roles in angiogenesis, blood vessel morphogenesis, blood vessel development, and vasculature development (**Fig. S3**). Disentangling the EC subpopulations responsible for different processes after a particular intervention would provide valuable biological information. After assigning putative identities, we sought to define the two clusters based on relative gene expression. Car4 was observed to be the most discriminating gene between the endothelial clusters, allowing cluster 2 to be defined as Car4-high ECs (**Fig. S4**).

### Car4-low ECs might be responsible for neutrophil recruitment

**Figure 5:**
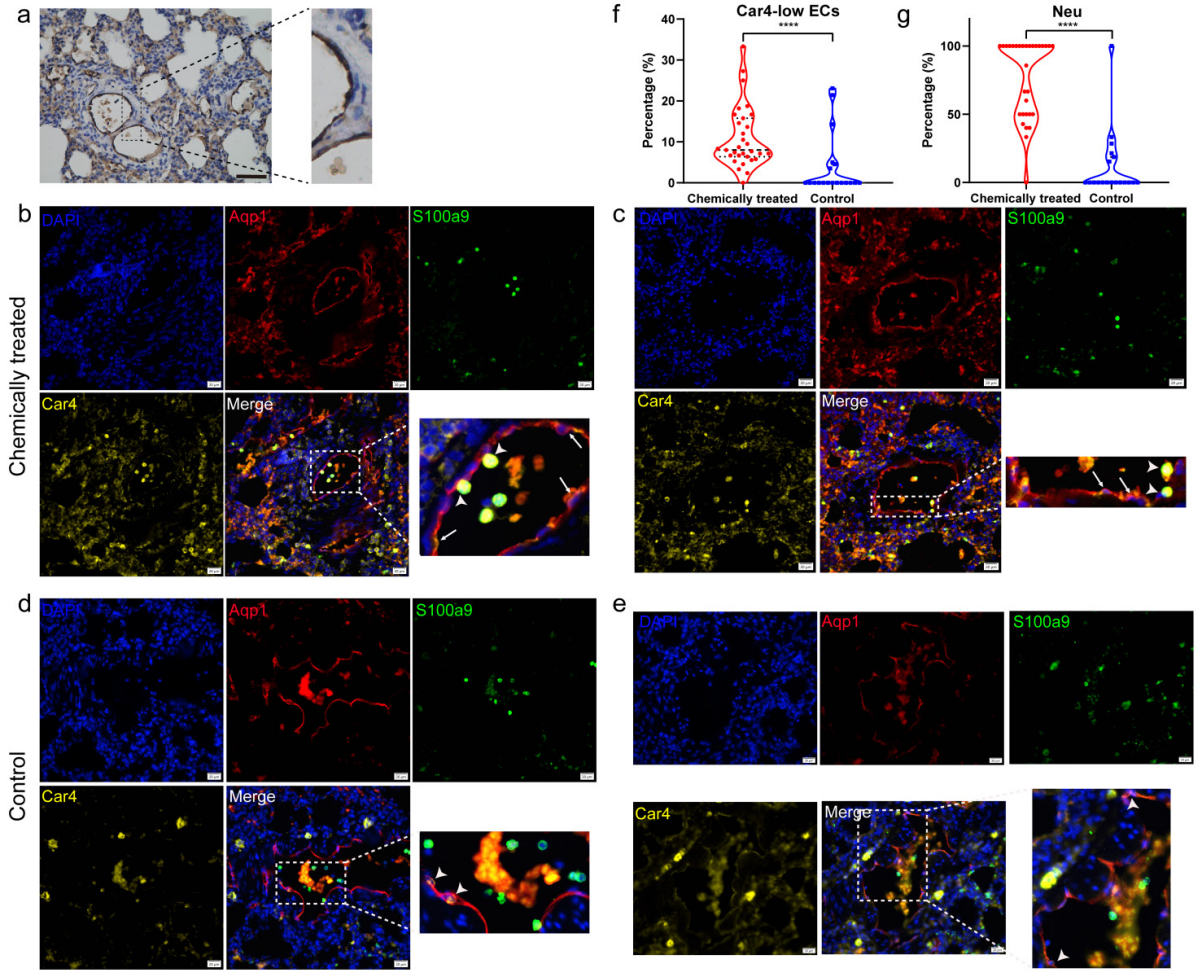
** Histological verification by immunohistochemistry and immunofluorescence staining. a** Immunohistochemical staining of Aqp1 as an EC marker in rat lung sections. The results represent those from three sections, with six pictures scanned for each section. Scale bar: 100 µm. **b, c** Immunofluorescence verification of the adhesion between neutrophils (S100a9) and Aqp1^+^/Car4^-^ ECs in inflamed tissues. DAPI: blue, Aqp1: red, S100a9: green, and Car4: yellow. Stubby arrows indicate adhesions between cells, and long arrows indicate Aqp1^+^/Car4^+^ ECs. Scale bar: 20 µm. **d, e** Immunofluorescence colocalization of Aqp1 and Car4 in ECs of normal tissues. Arrows indicate Aqp1^+^/Car4^+^ ECs. Scale bar: 20 µm. **f** Comparison of the number of Car4-low ECs interacting with neutrophils as a percentage of the total number of Car4-low ECs in blood vessels between the chemically treated group and the control group. **g** Comparison of the number of neutrophils adhering to Car4-low ECs as a percentage of the total number of neutrophils in blood vessels between the chemically treated group and the control group. The immunofluorescence results represent those from three sections, with nine pictures scanned for each section. EC: endothelial cell, Neu: neutrophil, ^****^P < 0.0001.

Nonimmune cluster 4 (Car4-low ECs) was further characterized in the subsequent analysis. The expression levels of the feature genes Upp1 and Lrg1 were particularly high in neutrophils (**Fig. 4e**). Hence, we speculated that Car4-low ECs might be involved in interactions with neutrophils. Neutrophil recruitment during inflammatory responses has been widely reported, and ECs are thought to play an important role in this process. To verify that Car4-low ECs exhibit inflammatory properties, we next inspected genes related to neutrophil recruitment and inflammatory response in Car4-low ECs and examined their expression in the two EC subsets. Lrg1 is expressed during neutrophil differentiation and has been demonstrated to be involved in cell adhesion and signal transduction 23. Many inflammation-related genes were identified in Car4-low ECs (**Fig. 4d**), such as the complement receptor C5ar1, inflammatory cytokine Il33, transmembrane immune signaling adaptor Tyrobp, proinflammatory chemokine Cxcl2, transcription factor Hmgb2, and Fc fragments of Ig receptor (Fcgr1a, Fcgr2a, Fcgr2b, Fcgr3 and Fcer1g). In line with the functional enrichment analysis, most cytokines, chemokines, and other immune-related genes were more expressed in Car4-low ECs than in Car4-high ECs (**Fig. S5**). Consistent with this observation, the key genes in Car4-low ECs identified by Cytoscape software overlapped, further implying the participation of Car4-low ECs in neutrophil recruitment (**Fig. 4f**). For histological verification, Aqp1 was verified as an EC marker in the corresponding tissue (**Fig. 5a**). Adhesion between neutrophils (S100a9, green) and Car4-low ECs (Aqp1^+^/Car4^-^, red) was observed in the inflamed sample (as shown by the stubby arrows). Car4-high ECs (Aqp1^+^/Car4^+^, red/yellow) were also found in the same blood vessel but were not involved in neutrophil interactions (as shown by the long arrows) (**Fig. 5b, c**). In the control group, almost no interactions between these two types of cells were observed (Aqp1^+^/Car4^+^ ECs are indicated by arrows) (**Fig. 5d, e**). Quantitatively, the percentages of Car4-low ECs (*P* < 0.0001) and neutrophils (*P* < 0.0001) that adhered to each other in blood vessels were significantly higher in the chemically treated group than in the control group (**Fig. 5f, g**).

## Discussion

With the deciphered transcriptomic profiles of rat lung tissues from the inflammation and control groups, our study provides new and high-resolution insights into lung inflammation. Inflammation-related changes in cell type composition and gene expression were observed at the single-cell level. Most importantly, we defined a unique subset, Car4-low ECs, and characterized its potential role in recruiting neutrophils to inflamed tissues, thereby expanding our understanding of endothelial heterogeneity in the lungs and shedding light on the cell-cell interactions underlying the inflammatory process.

The established rat model involving the intratracheal instillation of MCA and DEN enables the study of multistep lung carcinogenesis at the cellular and molecular levels. In this study, the pulmonary inflammation phase was observed in addition to lung tumor and precancerous lesions (including hyperplasia, metaplasia, and dysplasia). There have been several attempts to uncover the transcriptomic shift during pulmonary inflammation. As a transformative technology, scRNA-seq allows the determination of cellular diversity and the elucidation of cellular mechanisms. A total of 14 cell types were identified in our study. Comparative analysis revealed the effect of carcinogen stimuli on immunomodulation and highlighted the potential influence of this intervention on cell composition and gene expression patterns. Of note, examination of the nonimmune population revealed significant heterogeneity. The relative proportions of ECs, SMCs, Epi and Fib were dramatically elevated in rat with inflammation. The results of the correlation analysis further validated the differences between samples. In addition, the correlation coefficient of B cells was low, and the population of these cells was higher in the inflamed sample, as suggested by the enrichment of the immune effector process. Inflammation-related biological processes were enriched in neutrophils from chemically treated rat.

Nonimmune cells were further explored by reclustering. Two subpopulations of ECs with distinct roles were recognized, and cell type-specific characterization of biological functions was conducted. The identified EC subset with high Car4 expression appeared to play a specialized role in the regulation of angiogenesis, which was consistent with the scRNA-seq literature suggesting that Car4-high ECs are primed for vasculogenesis and blood vessel development 24. And Ellis *et al.* revealed that Car4 ECs possess a transcriptional signature suggestive of a contribution to alveolar angiogenesis in mouse lungs 25. Car4 was reported to be highly expressed in capillaries, suggesting that the identified subset might belong to capillary ECs 26, 27. Moreover, the results highlighted the key identity of Car4-low ECs as an independent, highly specialized subset dedicated to interactions with neutrophils. These cells might be Lrg1-high, and Lrg1-high ECs were reported to be involved in regulating cell adhesion 24; however, the difference in expression between EC subsets tended toward but did not reach significance (*P* = 0.059). It has been suggested that lung capillaries can be composed of both Car4-high and Car4-low ECs 25, and they were observed to share a common lumen in our study.

Among the top highly expressed genes in Car4-low ECs, Upp1 was revealed to be associated with immune and inflammatory responses 28. Lrg1 appears to function as a novel proinflammatory factor that is secreted upon neutrophil activation to regulate the immune microenvironment 29. Regarding the adhesion molecule Icam2, a previous study suggested its involvement in neutrophil extravasation 30. Cnn3 was reported to participate in cell adhesion. Collectively, the abundant expression of Upp1 and Lrg1 in neutrophils and the high levels of adhesion-related molecules observed in Car4-low ECs underscored their specific functional roles, which were enriched in the positive regulation of cytokine production, regulation of inflammatory response, neutrophil chemotaxis and migration. The process of neutrophil recruitment is triggered by changes on the endothelial surface in response to stimulation with inflammatory mediators, and this process involves a cooperative interplay among cytokines, chemokines, their receptors, and other immune-related genes 10. We next identified key genes with crucial roles in functional modulation. A series of Fc fragments of the Ig receptor, including Fcgr1a, Fcgr2a, Fcgr2b, Fcgr3 and Fcer1g, were demonstrated to be critical in Car4-low ECs. Fcgr is considered to be an active participant in neutrophil recruitment, and Fcgr2a and Fcgr3 contribute to enhanced adhesion and emigration 31, 32. Tyrobp, also known as DAP12, is primarily expressed on myeloid cells and is required for integrin-mediated neutrophil activation 33. The inflammatory receptor Trem1 was proven to signal through intracellular activation of the adaptor DAP12 34. With respect to Cxcl2, this potent neutrophil chemoattractant is responsible for neutrophil emigration 35 and can induce neutrophil recruitment into the lung of rats 36. The complement receptor C5ar1 was implicated in the initiation of neutrophil adhesion to the endothelium 37. Il33, an inflammatory cytokine abundantly expressed in ECs, is considered a critical regulator of chronic inflammation 38. Additionally, the transcription factors Egr1 and Hmgb2 have been reported to be involved in inflammatory regulation 39, 40. It was interesting to note that neutrophils in the inflamed tissue also stained positive for Car4, and Car4 expression in neutrophils was higher in the chemically treated group than in the control group (**Fig. S6**). Car4 has been reported to be expressed on activated granulocytes in murine lung inflammation 41, indicating that its expression level in neutrophils may be associated with cellular activation in response to inflammation. Furthermore, our observations, combined with the different functional enrichments of neutrophils in these two groups, suggested the potential presence of neutrophil subsets with diverse phenotypes. The presence of two separate lineages of neutrophils has been certified, and only a selective population of neutrophils with a proinflammatory role are recruited by the chemokine Cxcl2 from circulation to sites of injury 42, suggesting the feasibility of further mining the proinflammatory neutrophil subpopulation.

Evidences from relevant literature and histological verification have suggested the reliability of our findings and interpretations. However, our study also faces limitations. First, we primarily focused on restricted samples, which might introduce individual differences or contingencies. Thus, additional samples from each group are required to probe inter-sample heterogeneity. Second, the steps in the preparation of single cell suspensions, such as enzymatic dissociation, might lead to systematic deviation due to different degrees of damage to different cell types. The isolation procedures should be improved in the future by optimizing the sample processing time and raising the sensitivity for the detection of delicate cells. Third, although the number of loaded cells in this study was sufficient to support our analysis, the inclusion of more cells for sequencing would provide a more comprehensive cellular landscape of pulmonary inflammation. In terms of whether the EC subset was persistent or appeared at a particular stage during the inflammatory response, in-depth exploration would be significantly relying on pseudotime analysis and lineage tracing experiments. Moreover, the biological role of this subset is putative, and *in vitro* experiments are required as proof of concept.

Our results collectively provide insight into the mechanism underlying the interaction between Car4-low ECs and neutrophils and uncover inflammation-associated signatures at the molecular level, which might add dimension to the biological foundation of EC subsets. Continuous neutrophil recruitment may create an ideal inflammatory environment that promotes cell malignant transformation. The current study may set the stage for targeting specific subpopulations and inflammatory processes to alleviate pulmonary inflammation and may serve as a scaffold to facilitate future studies on the development of potential biomarkers and chemoprevention strategies for inflammation-related lung cancer. Single-cell analysis is expected to elucidate developmental processes and regulatory pathways, thereby enabling the mapping of transcriptional phenotypes to different subsets. We are dedicated to ascertaining the dynamic evolution process of multistep lung carcinogenesis at the single-cell level in subsequent research. A deeper understanding of the interactions between and molecular mechanisms underlying inflammation and lung carcinogenesis will enable interventions at the early stage of disease, which may offer more benefit to high-risk population.

## Methods

### Establishment of the rat lung carcinogenesis model

A total of 50 male Wistar rats (6 weeks old, 200 ± 10 g) (Dossy, Chengdu, China) were housed in a temperature- and humidity-controlled room with a light/dark cycle of 12 h and free access to food and water. All the animal surgical and experimental procedures were approved by the Ethics Board of Experimental Animals, West China Hospital, Sichuan University (Approval No. 20211552A).

MCA and DEN (Sigma-Aldrich, St. Louis, USA) were dissolved in iodized oil at 50 mg/ml and 100 μl/ml and incubated at 70 °C overnight. After the acclimation period, 35 rats were completely anesthetized with 3% sodium pentobarbital (30 mg/kg) by intraperitoneal injection; then, 0.1 ml of carcinogen-containing iodized oil was instilled into the trachea at the end of expiration. The same dose of iodized oil without any carcinogen was infused into another 15 rats as controls. Apart from the rats lost during anesthesia, intratracheal instillation, and subsequent feeding, 24 rats remained in the chemically treated group and 10 rats remained in the control group. The rats were euthanized by excessive anesthesia at 4, 6, 8, 10, and 12 weeks after instillation, and the lungs were bisected for tissue fixation and the preparation of single cell suspensions.

### H&E staining

Pieces of lung tissues were fixed in 4% formaldehyde in phosphate buffer for 24 h, embedded in paraffin, cut to a thickness of 4 μm, and then routinely stained with hematoxylin and eosin (H&E). H&E-stained slides were microscopically examined, and pathological classification was performed by experienced pathologists.

### Preparation of single cell suspensions

Single cell suspension of lung tissue was obtained by mechanical dissociation and enzymatic digestion. Lung tissue was quickly washed in PBS, placed in a 5-ml Eppendorf tube, and then minced into pieces as small as possible using a sterile surgical scalpel. The entire process was performed on ice. The tissue fragments were then digested with 2 mg/mL collagenase I (Washington, Lakewood, USA) and 1 mg/mL collagenase IV (Washington) in Hanks' balanced salt solution (HBSS, GIBCO, NY, USA) for 45 minutes at 37°C. The digested cells were successively filtered through 70-μm and 30-μm cell strainers (Corning, NY, USA), and red blood cells (RBCs) were removed with RBC lysis buffer (Invitrogen, Carlsbad, USA). After centrifugation, dead cells were removed from the cell suspension with a Dead Cell Removal Kit (Miltenyi Biotec, Bergisch Gladbach, Germany), and live cells were sorted into PBS with 0.04% BSA for subsequent scRNA-seq.

### Library construction and single-cell RNA sequencing

The single cell suspensions were immediately processed and loaded into a Chromium microfluidic chip of the Single Cell 3' Chip Kit (10x Genomics, CA, USA). The standard 10x Chromium Single Cell 3' protocol was followed for library preparation. Briefly, cells with specific 10x barcodes and unique molecular identifiers (UMIs) were partitioned into nanoliter-scale Gel bead-in-EMulsions (GEMs) using 10x GemCode Technology. RNA from the barcoded cells was subsequently reverse transcribed, and sequencing libraries were constructed with a Chromium Single Cell 3' reagent kit (10x Genomics). Full-length cDNA was purified using DynaBeads MyOne Silane Beads (Thermo Fischer Scientific, Waltham, USA) and amplified by polymerase chain reaction (PCR). Enzymatic fragmentation and size selection were conducted by SPRIselect Reagent (Thermo Fisher Scientific). After adaptor ligation and sample index PCR, the cDNA libraries were sequenced on an Illumina NovaSeq 6000 according to the manufacturer's instructions.

### Processing and analysis of scRNA-seq data

Raw scRNA-seq data were processed by Cell Ranger provided by 10x Genomics. The cell population was grouped by K-means-based clustering in Seurat based on the gene expression profile of each cell. The downstream single-cell analyses were performed by Cell Ranger and Seurat with default parameters unless otherwise specified. According to quality control criteria, abnormal cells in the datasets were filtered based on their gene expression distribution. A gene that was expressed in more than five cells was considered expressed. Cells expressing fewer than 200 genes or more than 10% mitochondrial genes were removed. The data were then log-normalized for subsequent analysis. Through principal component analysis (PCA) for dimensionality reduction, similarity between cells was observed. Clustering analysis was visualized with t-SNE for the top 17 principal components. Gene Ontology enrichment analysis was conducted using Metascape and the clusterProfiler R package. The protein-protein interaction (PPI) network was generated from the STRING database, and the Cytoscape CytoHubba plug-in was used to identify hub genes in the complex network.

### Immunohistochemical staining

Paraffin-embedded tissue sections were deparaffinized with xylene in a gradient alcohol series and then rehydrated in water. The slides were immersed in citrate buffer (pH = 9.0), and antigen retrieval was performed by microwave heating. Endogenous peroxidase activity was blocked by incubation with 3% hydrogen peroxide, and 10% normal goat serum was used to block nonspecific proteins. The samples were incubated with a primary antibody against Aqp1 (Santa Cruz Biotechnology, Santa Cruz, CA) in a humidified chamber at 4 °C overnight. After incubation with a secondary antibody at 37 °C for 1 h, the signal was detected by streptavidin-horseradish peroxidase and diaminobenzidine. The slides were then lightly counterstained with hematoxylin, dehydrated in a graded alcohol series, cleared in xylene, and covered. The degree of Aqp1 positivity and the proportion of Aqp1-positive ECs in the vascular area were determined by experienced pathologists to evaluate whether Aqp1 could be used as an EC marker. The results represent those from three sections, with six pictures scanned for each section.

### Immunofluorescence histochemical staining

Goat serum (10%) was used to block the binding of nonspecific proteins to the sections. Primary antibodies were diluted and incubated with the sections overnight at 4 °C: anti-Aqp1 (Santa Cruz Biotechnology), anti-S100a9 (Proteintech, Rosemont, USA), and anti-Car4 (R&D Systems, Minneapolis, USA). The slides were then washed 3 times with PBS and incubated with secondary antibodies (Jackson ImmunoResearch Laboratories, West Grove, USA) for 1 h. The nuclei were counterstained with DAPI (4′,6-diamidino-2-phenylindole, Sigma-Aldrich). Images were captured with an IX83 fully automatic inverted fluorescence microscope (Olympus, Tokyo, Japan). Adhesion of neutrophils (S100a9) to Aqp1^+^/Car4^-^ ECs or Aqp1^+^/Car4^+^ ECs was evaluated by experienced pathologists. For quantification, the numbers of adherent Car4-low ECs and neutrophils per blood vessel were counted. Doublets and triplets were split by considering signal over the average size of a cell. Data were depicted as percentages of Car4-low ECs (number of Car4-low ECs interacting with neutrophils/total number of Car4-low ECs in the blood vessel) and neutrophils (number of neutrophils adhering to Car4-low ECs/total number of neutrophils in the blood vessel) per field. The results represent those from three sections, with nine pictures scanned for each section.

### Statistical analysis

The Mann-Whitney-Wilcoxon test was applied to analyze data between two groups. A *P* value less than 0.05 was considered statistically significant. Data were visualized with the ggplot2 R package and GraphPad Prism 8.0.

## Supplementary Material

Supplementary figures.

## Abbreviations

EC: endothelial cell
MCA: 3-methylcholanthrene
DEN: diethylnitrosamine
scRNA-seq: single-cell RNA sequencing
CCA: canonical correlation analysis
t-SNE: t-distributed stochastic neighbor embedding
NK: natural killer
Neu: neutrophil
Mono: monocyte
DC: dendritic cell
Macro-alv: alveolar macrophage
Macro-inter: interstitial macrophage
Fib: fibroblast
SMC: smooth muscle cell
Epi: epithelial cell
DEG: differentially expressed gene
GO: gene ontology
H&E: hematoxylin and eosin
HBSS: Hanks' balanced salt solution
RBC: red blood cell
UMI: unique molecular identifier
GEM: gel bead-in-emulsion
PCR: polymerase chain reaction
PCA: principal component analysis
PPI: protein-protein interaction
