# Supplementary material for: Single-cell RNA Sequencing Uncovered the Involvement of an Endothelial Subset in Neutrophil Recruitment in Chemically Induced Rat Pulmonary Inflammation
Source: Int J Med Sci. 2022 Mar 28;19(4):669–80. doi: 10.7150/ijms.67806 (PMC9108403; doi:10.7150/ijms.67806)
Supplement: Supplementary file 1 — Supplementary figures. [file ijmsv19p0669s1.pdf]

Single-cell RNA Sequencing Uncovered the Involvement of an Endothelial Subset in Neutrophil Recruitment in Chemically Induced Rat Pulmonary Inflammation

Supplementary figures:

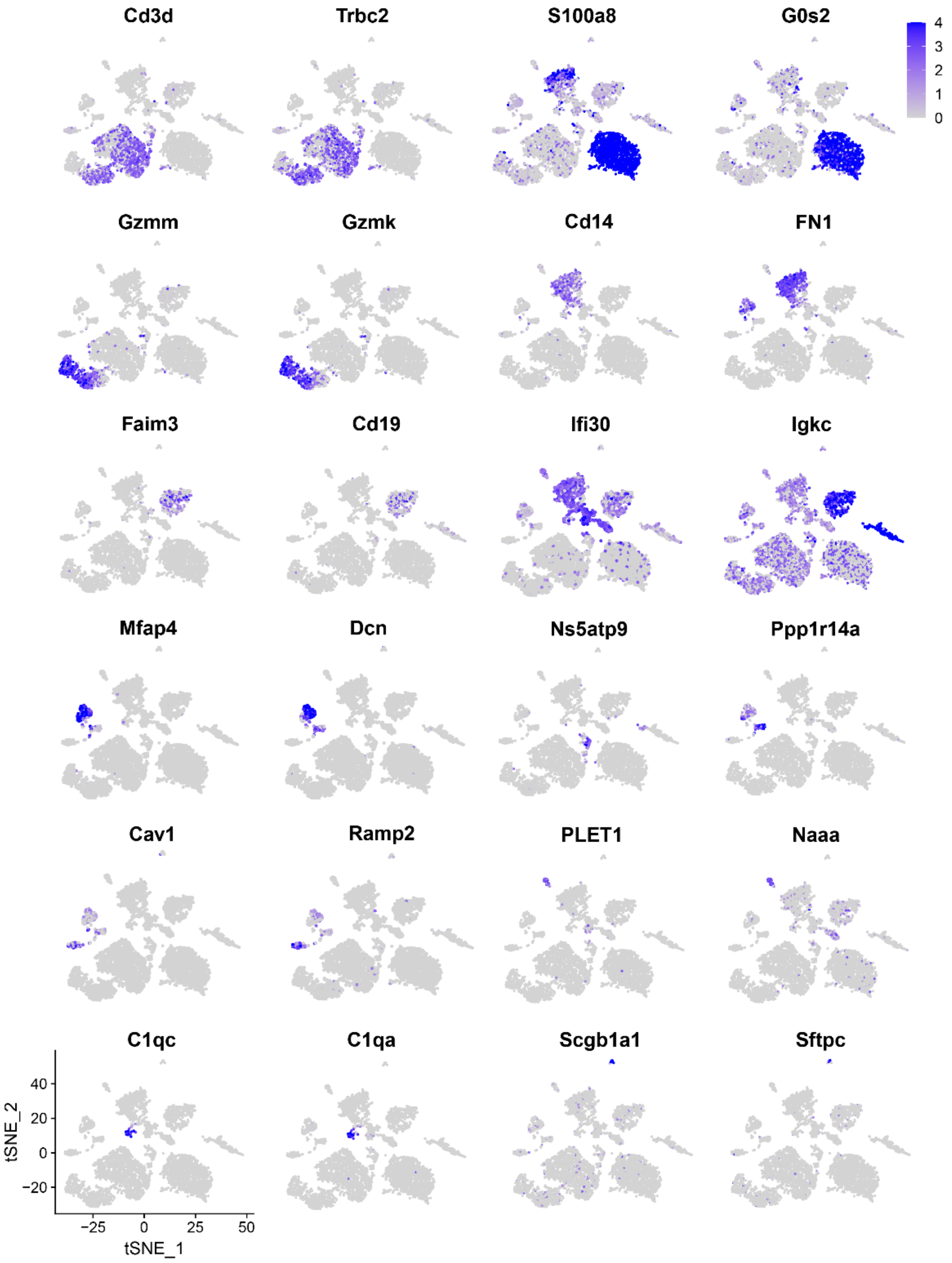

Fig. S1 Feature plots of marker genes in each cell type, as Fig. 2c. Each dot represents a single cell, and the color gradient represents the normalized gene expression.

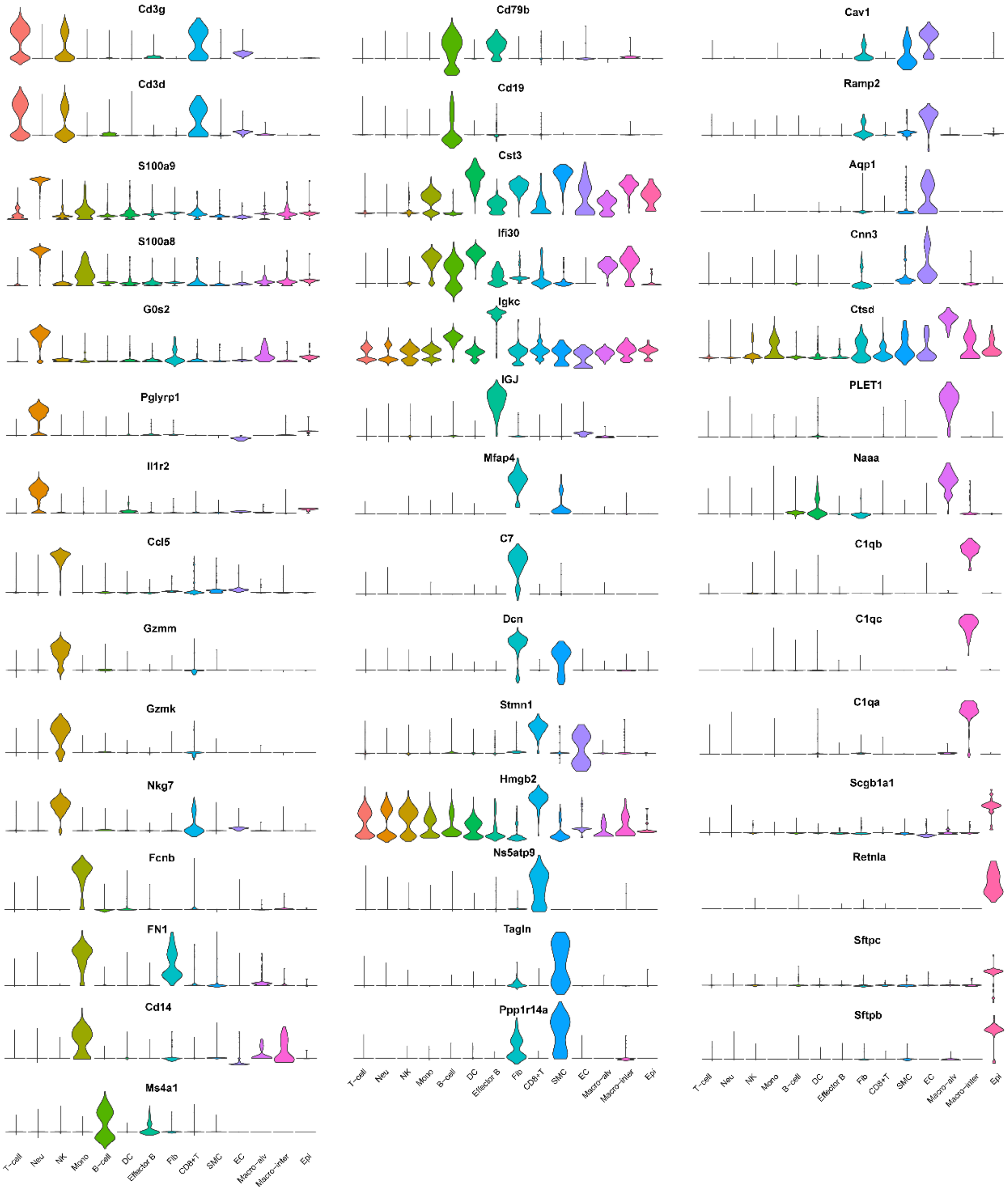

Fig. S2 Violin plots of selected markers of each cell type showing their gene expression levels.

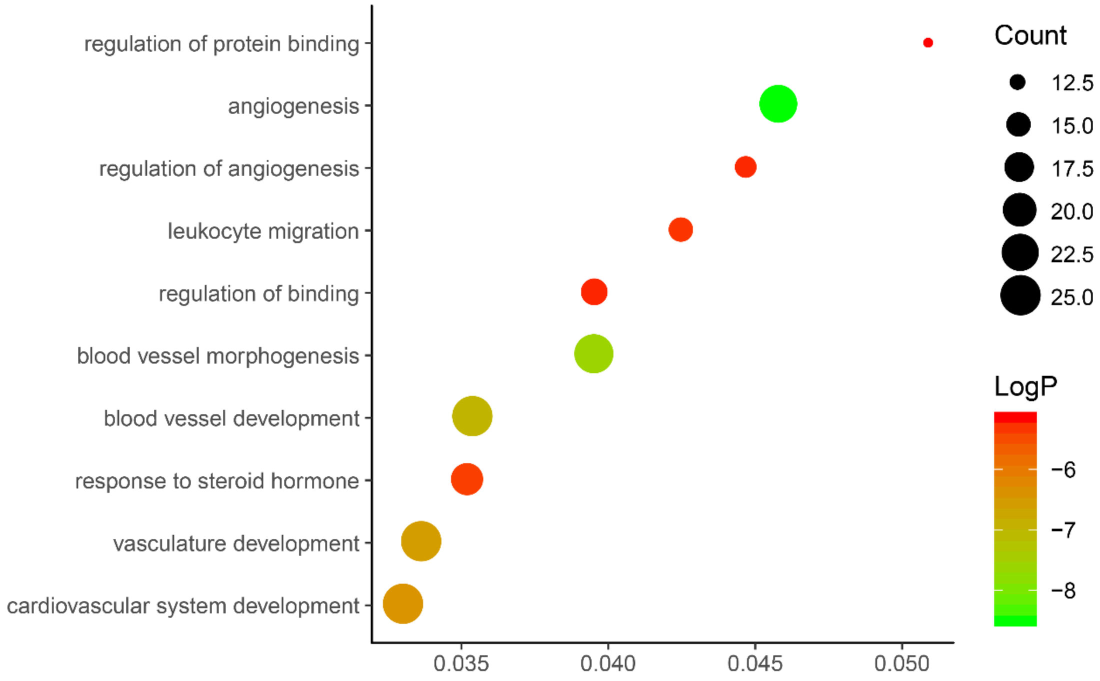

Fig. S3 GO (biological process) terms enriched in nonimmune cluster 2. Top 10 GO terms are ordered on the y-axis. X-axis represents the gene ratio. The size of dot represents the gene count. The color gradient of dot represents the log *P* of each enriched GO term.

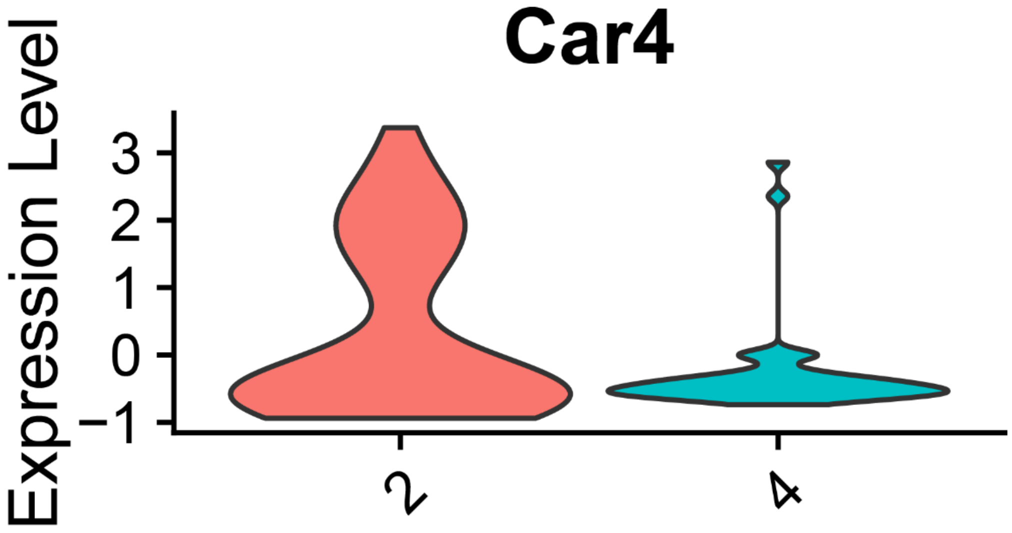

Fig. S4 Violin plot shows the expression level of Car4 between nonimmune cluster 2 and cluster 4.

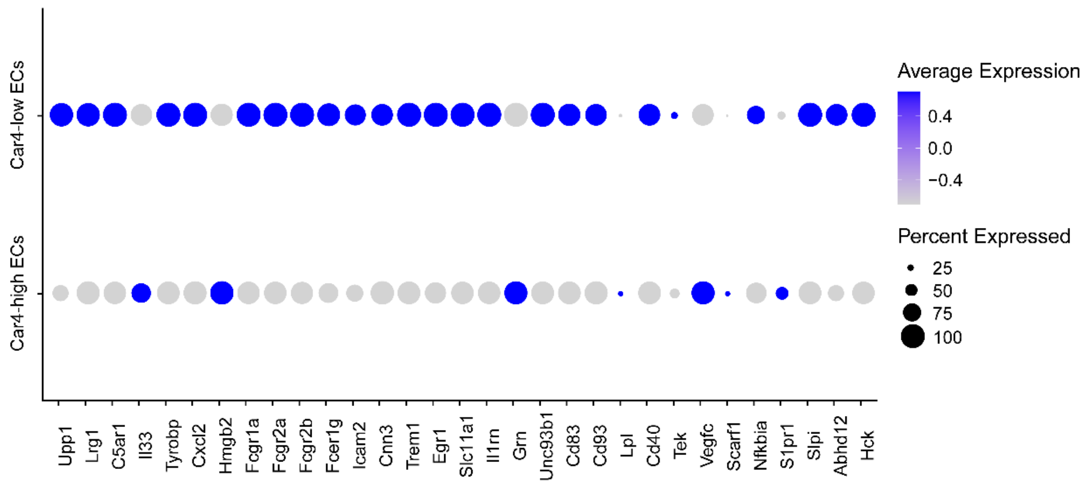

Fig. S5 Dot plot shows the expression of genes related to neutrophil recruitment and inflammatory response in the two EC subsets.

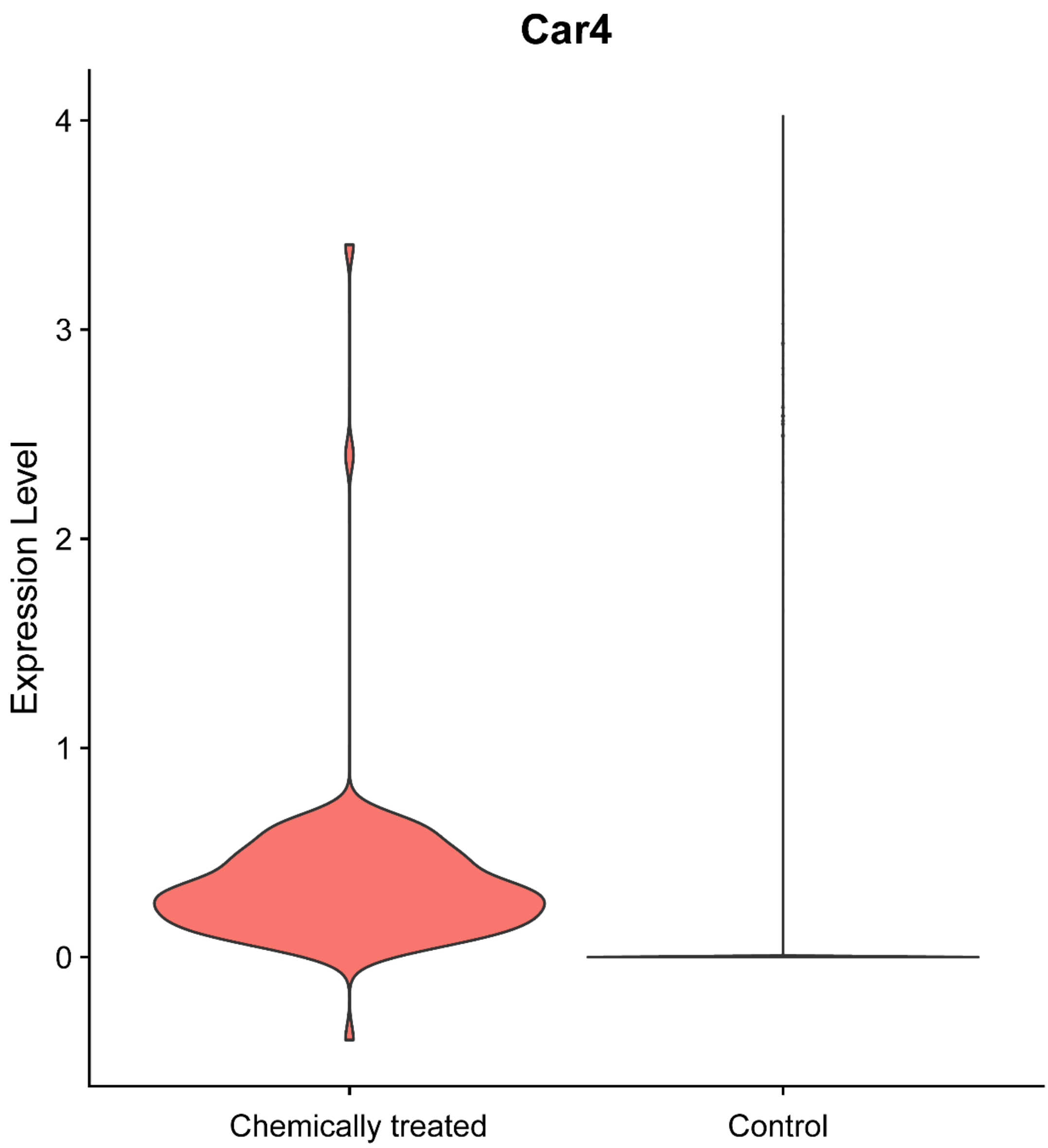

Fig. S6 Violin plot shows Car4 expression in neutrophils between chemically treated and control groups.
